# Synthesis of Thiazolo[5,4-*d*]thiazoles in an Eco-Friendly L-Proline–Ethylene Glycol Mixture

**DOI:** 10.3390/molecules30040938

**Published:** 2025-02-18

**Authors:** Thiên Thuý Trang Nguyễn, Jean-François Longevial, Stéphanie Hesse

**Affiliations:** LCP-A2MC, Université de Lorraine, 1 Boulevard Arago, F-57000 Metz, France; thien-thuy-trang.nguyen@univ-lorraine.fr (T.T.T.N.); jean-francois.longevial@univ-lorraine.fr (J.-F.L.)

**Keywords:** thiazolo[5,4-*d*]thiazole, deep eutectic solvent, L-proline-based DES, microwave, zero-VOC strategy, sodium metabisulfite

## Abstract

The hazardousness of solvents used in synthetic organic chemistry is well established. In this context, it is relevant to search for safer and greener alternatives. Within the last decades, deep eutectic solvents have been considered as possible and promising alternatives. Consequently, this study aims at using deep eutectic solvents to synthesize an emerging class of heteroaromatic compounds named thiazolo[5,4-*d*]thiazoles, for which interest is growing in the field of organics, electronics, and biology. To address this challenge, we developed a straightforward synthetic protocol consisting of condensing dithiooxamide and aromatic aldehyde in deep eutectic solvents to yield the desired thiazolo[5,4-*d*]thiazole without further purification. The first hit was obtained with the well-known L-proline:glycerol (1:2) mixture at 130 °C. However, dithiooxamide is degraded under these conditions, leading to the formation of impurities that may arise from the consequent amount of reactive L-proline. Reaction conditions were optimized by modifying the deep eutectic solvent nature and proportions, applying various temperatures, changing the activation and heating source, or adding auxiliary oxidants. As a consequence, eight thiazolo[5,4-*d*]thiazoles were synthesized in equal or better yields (20 to 75%) than the reported procedure under safe and eco-friendly conditions in a mixture of L-proline and ethylene glycol (1:50) with sodium metabisulfite at 130 °C for one hour.

## 1. Introduction

**Figure 1:**
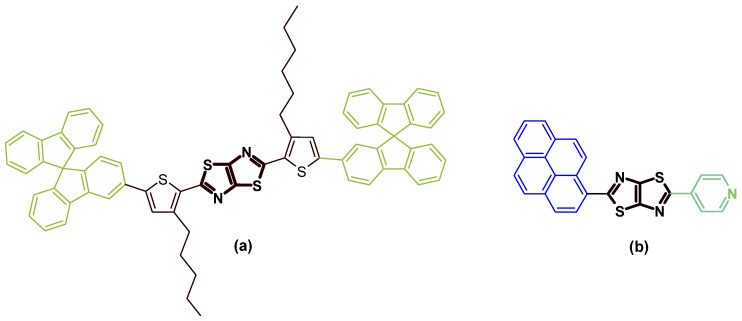
(**a**) Example of symmetric TzTz. (**b**) Example of asymmetric TzTz. The TzTz backbone is bolded in both cases.

In the last few decades, small organic molecular dyes have attracted great interest due to their wide applications in optoelectronic devices such as organic light emitting diiodes (OLEDs) or photovoltaics [1,2,3,4]. Among these small organic molecular dyes, thiazolo[5,4-*d*]thiazoles (TzTzs) which are formed by two fused coplanar thiazole rings are particularly interesting [5]. This rigid backbone presents an extended π-conjugated system with a high oxidative stability thanks to the electron-deficient nature of the sp^2^ nitrogen [6]. Moreover, tuning of their electronic properties can be achieved by modifying their symmetry. Indeed, TzTzs can be qualified as symmetric with identical moieties on both sides or asymmetric when bearing different ones. As an example, symmetric TzTzs with a spirobifluorene moiety have been described in 2023 for their applications in electroluminescent devices [7] (Figure 1a), whereas an asymmetric TzTz incorporating a pyrene (donor) and a pyridine (acceptor) (Figure 1b) was used as a push–pull system with promising applications in bio-imaging [8].

Due to their wide range of applications, TzTzs are increasingly studied even though the interest in this moiety is relatively recent. Described for the first time by Ketcham et al. in 1960 [9], there were only eleven papers dealing with this kind of compound in 2009. From this year forward, a great interest in TzTzs has been observed, going from 9 papers per year between 2010 and 2017 to 27 papers per year since 2018 (Supporting Information, Figure S1).

**Scheme 1:**
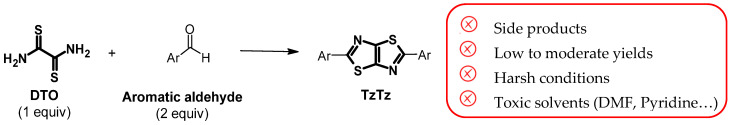
Classical way of synthesis of TzTz from 1 equiv. of **DTO** and 2 equiv. of aldehyde.

Even if there is a great interest in TzTz-based materials, the synthesis of those compounds still generally relies on the well-known Ketcham method [9], while exploring other conditions is only marginally done. Actually, the synthesis of TzTzs seems quite easy as they can be obtained in a single step with a double condensation reaction between dithiooxamide (**DTO**) and an aromatic aldehyde (Scheme 1). However, one serious drawback is their laborious isolation and purification as the reaction often leads to resinification and formation of side products, giving low to moderate yields of TzTzs [6]. Moreover, TzTzs are generally poorly soluble and are sometimes difficult to characterize [10].

Historically, reactions were carried out in neat conditions using a large excess of aldehydes [9,11]. Later, the condensations were realized in high boiling point solvents by applying harsh conditions (extended times and high temperatures). In most cases, harmful solvents such as DMF (the most often used) [6,12,13,14], pyridine [15], nitrobenzene, [6,13,16,17,18], phenol [19,20] or chlorobenzene [21] are used. Only few reports of TzTz synthesis state the use of non-toxic solvents such as ethanol [22,23].

Since the development of the green chemistry principles exposed in 1998 by Anastas and Warner [24], the scientific community is searching for more environmentally friendly protocols for the synthesis of compounds of interest. In this context, many efforts were devoted to the search of greener and safer solvents. Particularly, deep eutectic solvents (DESs) are considered as a promising emerging class of green solvents [25] since their first description by Abbott in 2003 [26]. Indeed, they generally display remarkable advantages, such as non-flammability, low volatility, chemical and thermal stability, and recyclability (depending of course on the components chosen). Moreover, the creation of DESs only involves the formation of a hydrogen bond network between a H-bond donor (HBD) and a H-bond acceptor (HBA) obtained by heating the two components at moderate temperature. Depending on the individual components chosen, the properties of DESs, such as pH, viscosity, and ionic conductivity, can be modulated according to synthetic needs [25]. Finally, DESs were also recently evaluated for their biodegradability and their environmental impacts [27,28,29]. The most common DESs used in organic synthesis are based on choline chloride (CC); however, others based on betaine or L-proline are also very powerful [30,31,32,33,34]. As an example, we recently developed a zero VOC strategy using a L-proline:glycerol DES in a 1:2 molar ratio (Pro:Gly (1:2) DES) to perform Knoevenagel reactions on rhodanine, thiazolidine-2,4-dione, and barbituric acid [35,36].

That is the reason why our project focuses on developing an environmentally friendly synthetic method for symmetrical TzTzs. In this study, this approach aims at answering the following commonly observed bottleneck associated with TzTz synthesis: (i) the use of greener and safer solvents, (ii) the reduction of reaction times by using alternative activation sources such as ultrasonic or microwave activation, and (iii) the goal of minimizing or eliminating the need for intensive purification using methods such as column chromatography.

## 2. Results and Discussion

**Scheme 2:**
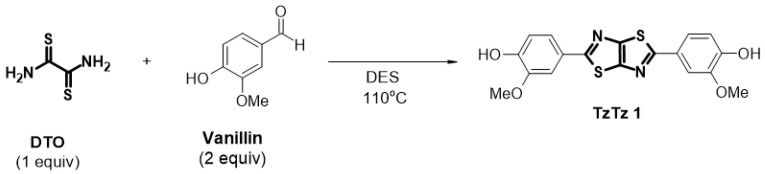
Synthesis of TzTz **1** in DES.

As mentioned above, our principal objectives were to synthesize TzTzs in a DES and to avoid a purification step using column chromatography. Consequently, we envisage a work-up procedure in which the desired product precipitates in pure form upon the simple addition of water. With this in mind, we started our study with a model reaction allowing the formation of TzTz **1**. Indeed, TzTz **1** is a particularly interesting compound not only because of its potential properties as a chemosensor [15], but also because it can be synthesized from vanillin, a bio-based aromatic aldehyde [37]. Therefore, in a first attempt, we tried to transpose the synthesis of TzTz **1** starting from vanillin and dithiooxamide **DTO** in two of the most classical DESs, i.e., CC:Gly (1:2) and CC:urea (1:2) (Scheme 2).

We carefully observed the reaction media and monitored the reaction progress using thin layer chromatography. The expected product was formed, but conversion was rather slow in both above-mentioned DESs as it needed 24 h at 110 °C for a total consumption of starting material (Table 1, entries 1–2). Alternatively, we recently successfully used a L-proline:glycerol (1:2) DES to perform a Knoevenagel reaction on rhodanine and form 5-arylidenerhodanine derivatives in a zero VOC strategy [35,36]. To our delight, performing the reaction in the Pro:Gly (1:2) DES was much faster as formation of TzTz **1** was achieved within an hour, consequently constituting a very promising result (Table 1, entry 3). However, using this Pro:Gly (1:2) DES, we observed a very rapid color change of the reaction media that made us think about a plausible degradation process, even though TzTz **1** was proved to be formed as evidenced by the ^1^H NMR of the crude material (Supporting Information, Figure S2). Therewith, we also noticed the presence of many spots on the TLC plates that can correspond to either intermediates or degradation products.

**Figure 2:**
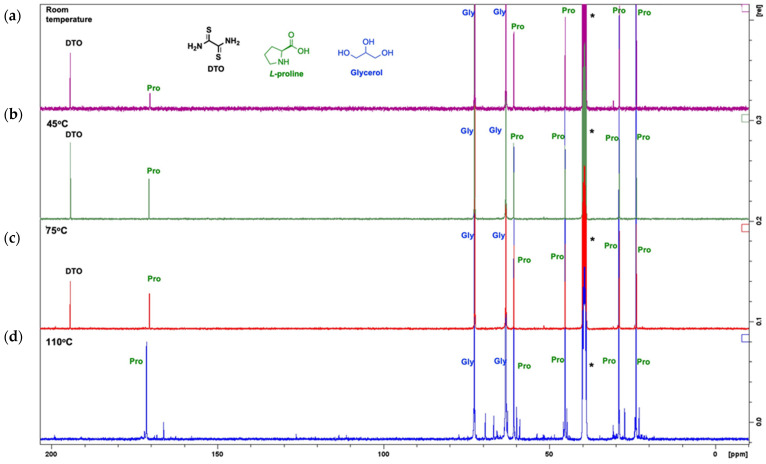
^13^C{^1^H} NMR study of **DTO** stability in the Pro:Gly (1:2) DES at different temperatures: (**a**) room temperature, (**b**) 45 °C, (**c**) 75 °C and (**d**) 110 °C. * corresponds to DMSO signal.

At first, we suspected a possible degradation of dithiooxamide under these reaction conditions. We therefore conducted a ^13^C{^1^H} NMR study of **DTO** stability in the Pro:Gly (1:2) DES at different temperatures (Figure 2). This study revealed that **DTO** is stable in Pro:Gly (1:2) at ambient temperature but also after 1 h heating at 45 °C as the characteristic signal of the *C*=S is clearly present at δ = 194.4 ppm (Figure 2a,b). This signal is still present after 1 h heating at 75 °C; however, we could observe new signals that may indicate the start of **DTO** degradation (Figure 2c). Finally, after 1 h of heating at 110 °C, the characteristic *C*=S **DTO** signal disappeared and many unidentified signals arose in the 20–80 ppm region (Figure 2d). These results clearly indicate **DTO** degradation under high temperature conditions in the Pro:Gly (1:2) DES.

We also conducted additional tests to study the stability of **DTO**. As control experiment, we studied the stability of **DTO** in DMF at 110 °C for one hour to have a closer understanding of its degradation process; its ^13^C{^1^H} NMR spectrum showed the presence of the characteristic *C*=S **DTO** signal at δ = 194.4 ppm (Supporting Information, Figure S3) and consequently its stability under these conditions. In parallel, we tested the interaction between **DTO** and L-proline by performing a reaction of these two compounds in DMF. After 1 h of heating at 110 °C, some by-products are formed. From this observation, we believe that the reaction between L-proline and **DTO** in DES is one of the sources of the observed impurities during TzTz formation.

We then performed a reaction with a slight excess of **DTO** (1.2 equivalent instead of 1 equivalent) to offset its degradation in the DES at 110 °C. Unfortunately, this slight **DTO** excess did not match the expected result as TzTz **1** is still contaminated with the same amount of impurities after 1 h.

As the degradation of **DTO** in DES occurs at high temperatures, we thought that lowering the temperature would prevent the impurity from forming. A first attempt at 45 °C revealed that vanillin was fully consumed, however, without any TzTz **1** formation, even after 24 h (Table 2, entry 2). Monitoring of the latter reaction using TLC nevertheless revealed the formation of an unidentified intermediate together with some impurities. Performing the reaction at 75 °C lead to total consumption of vanillin and a disappearance of the unidentified intermediate after 5 h (TLC monitoring) (Table 2, entry 3). Unfortunately, heating at 75 °C also led to the formation of impurities as can be seen from the ^1^H NMR spectrum of the crude. Finally, we tried to conduct the reaction by applying a temperature gradient: (i) heating at 45 °C for 4 h (allowing the formation of the intermediate identified on TLC without the degradation of **DTO**) and then (ii) increasing the temperature to 110 °C for 1 h (trying to promote the oxidation process that seems to only occur above 75 °C). Once again, the later strategy did not permit improvements in the purity of crude product.

As we believed that the reaction between **DTO** and L-proline contained in the DES is one of the sources of the observed impurities and given that the DES Pro:Gly (1:2) is quite viscous at ambient temperature and therefore not very easy to handle, we decided to decrease the L-proline amount by using other ratios of Pro:Gly for this model reaction. Performing the reaction in Pro:Gly (1:4) and Pro:Gly (1:40) clearly furnished a cleaner crude (Supporting Information, Figure S4). Furthermore, we though that lowering the viscosity of the DES should be beneficial. We thus tried to use L-proline:ethylene glycol DES for this reaction in 1:4, 1:40, and 1:50 ratios. Finally, Pro:EG (1:50) gave the cleanest crude product (Supporting Information, Figure S4) and allowed for the formation of nearly pure TzTz **1** in 8% yield after 1 h heating at 130 °C.

As L-proline is present in very small quantities in this latter DES, it may be questioned whether the name DES is still appropriate. Indeed, the definition of a DES may certainly need to be reviewed in the future; however, for now, the term DES includes all associations of HBAs and HBDs with different ratios ranging from (1:2) to (1:90) [30]. With this in mind, we performed a control experiment by reacting vanillin and **DTO** in pure ethylene glycol. Surprisingly, no TzTz **1** was formed, clearly indicating the key role of L-proline in this reaction. We extended our reasoning by running the same experiment with a catalytic amount of L-proline in ethylene glycol (5 mol% relative to DTO, corresponding to Pro:EG (1:1300)). Under those conditions, TzTz **1** was formed, but the crude was less clean than when the reaction was performed in Pro:EG (1:50) (Supporting Information, Figure S5).

To better understand this observation, we also realized a ^1^H NMR spectrum of L-proline alone in DMSO-*d*_6_ at 0.0173M and a ^1^H NMR spectrum of Pro:EG (1:50) in DMSO-*d*_6_ (with the same concentration in L-Proline). By comparison (Supporting Information, Figure S6), the chemical shifts of the signal associated with L-proline alone are different from those belonging to the L-proline in the Pro:EG (1:50) mixture. We can therefore suppose that interactions between L-proline and EG exist and may favor our reaction.

As said before, our goal was to find a clean and eco-friendly process to synthesize TzTz. That means that we aimed to avoid excess use of aldehyde as well as toxic solvent for both the reaction and work-up. We expected to obtain a pure TzTz directly by quenching the reaction upon the addition of water as it provokes the precipitation of the product, which can be isolated by a simple filtration. Finally, we wanted to avoid purifications and especially column chromatography. At this stage, we have improved the safety by using a Pro:EG (1:50) mixture, but we still needed to improve the purity of the crude and above all improve the yield.

As a consequence, we turned our attention to the TzTz formation mechanism. This may help us to identify a crucial step. Indeed, several publications studied the Ketcham reaction mechanism [6,38], and it has been proven that this reaction has to be conducted under air to allow spontaneous dehydrogenation to give the final aromatic product. To facilitate this dehydrogenation step, an additional oxidant is sometimes added in the reaction. For example, Dessi et al. used chloranil and DDQ [16], whereas Papernaya et al. employed SeO_2_ [39]. We were particularly interested in safe and non-toxic mild oxidants that would not lead to the formation of by-products difficult to separate. We turned our attention to Na_2_S_2_O_5_ and K_2_S_2_O_8_, which are often used in heterocyclic synthesis to facilitate aromatization and the formation of benzothiazole or benzimidazole rings [40,41,42], and consequently decided to perform our model reaction in the presence of those two mild oxidants.

While potassium persulfate (K_2_S_2_O_8_) did not improve the purity of the crude, sodium metabisulfite did. To our delight, performing the reaction in the presence of 1.1 equiv. of Na_2_S_2_O_5_ for 1 h at 130 °C in the previously identified Pro:EG (1:50) mixture gave a clean TzTz **1** that did not need any further purification (Supporting Information, Figure S7). A satisfying yield of 75% was obtained (Table 3, entry 1). Moreover, an excellent yield of 92% was also obtained when using microwave irradiation (25 min at 130 °C). In our search of alternative activation sources, we have also realized the reaction under ultrasonic irradiation. Under these conditions, TzTz **1** was only scarcely formed perhaps because the temperature reached in the reaction media was only 75 °C. Having those optimized conditions in mind, we extended this methodology to other aldehydes. Using isovanillin, TzTz **2** was synthesized in good yields of 70% and 75% for classical heating and microwave irradiation, respectively (Table 3, entry 2). TzTz **3** was obtained from catechol in quantitative yields using classical heating (Table 3, entry 3). *p*-Hydroxy- and *m*-hydroxybenzaldehydes also gave satisfying yields under classical heating (72% and 85%, respectively), whereas disappointing yields were obtained for TzTz **4** and **5** under microwave irradiation (Table 3, entries 4–5). In those latter reactions under microwave heating, we have noticed the formation of impurities in a greater amount compared with conventional heating, making the TzTzs hard to isolate without further purification.

TzTz **6** is of particular interest as SNAr reactions could be considered. For example, 2,5-bis(4-fluorophenyl)thiazolo[5,4-*d*]thiazole unit **6** was incorporated in a polymer used as membrane [45]. TzTz **7** constitutes an interesting ligand for the formation of complexes [16] and was obtained with a satisfying 66% yield under our eco-friendly conditions. TzTz **8** can be used in palladium-catalyzed [46] or copper-catalyzed [47] cross-coupling to access more complex TzTzs. Finally, we also tested the reactivity of aldehydes containing a highly electro-withdrawing substituent (2-nitro- and 3-nitrobenzaldehydes). Unfortunately, the formed TzTz could not be isolated with satisfactory purity, which therefore constitutes a limitation of this new protocol.

In conclusion, we have found eco-friendly conditions to synthesize the very interesting TzTz moiety. We have demonstrated the importance of the DES choice on the rate of conversion of starting material (L-proline-based DES allowed a faster conversion than choline chloride-based DES). We have shown the impact of the DES on the purity of the crude (among the 8 mixtures tested, the Pro:EG (1:50) mixture gave the cleanest crude product). Of note, we have highlighted the importance of adding Na_2_S_2_O_5_ to the reaction to favor the dehydrogenation step (yield of TzTz **1** formation was increased from 8% to 75% upon addition of Na_2_S_2_O_5_). Moreover, these results were confirmed on another TzTz. Specifically, TzTz **2** was synthesized with 31% and 70% yields in the absence and presence of the additional oxidant, respectively. Finally, the majority of the TzTzs synthesized in this study were obtained under more eco-friendly conditions, with yields equivalent or superior to those reported in literature using harsh and hazardous solvents.

## 3. Materials and Methods

All reactions were routinely checked using TLC analysis on an Alugram SIL G/UV254 (Macherey-Nagel, Hoerdt, France) with spots visualized with UV light. The ^1^H and ^13^C{^1^H} NMR spectra were measured on an AC Bruker 400 MHz spectrometer (Bruker, Wissembourg, France) in DMSO-*d*_6_; chemical shifts are reported in parts per million (ppm). All coupling constants (J) are given in Hz.

TzTz **2** was analyzed using laser desorption ionization (LDI) FT-ICR MS in negative-ion mode. The solid sample was deposited on a MALDI stainless-steel target to be then desorbed and ionized using a 355 nm wavelength laser, with a 2 mm spot diameter and 1000 Hz frequency. Ions generated over the 100 laser shots were stored in a hexapole before being transferred to the ICR cell. Laser power was optimized at 7.8% to detect MS signals limiting the fragmentation and recombination phenomenon.

Both ion source and instrument parameters were optimized using FTMS-Control V2.3.0 software (Bruker Daltonics, Wissembourg, France). Prior to acquisition, the mass spectrometer was externally calibrated, and Ithe CR detection cell was shimmed and gated using the direct introduction of a 0.1 mg/mL sodium trifluoroacetate solution. Mass spectra resulted from the accumulation of 20 scans over a *m*/*z* 107.5–1500 range and with a 4 megaword time-domain.

At *m*/*z* 370, a mass resolution of 500,000 was achieved. Molecular formulae were assigned using SmartFormula Editor (Bruker Daltonics) with a ± 1 ppm mass accuracy window. A theoretical mass spectrum, with the isotopic distribution, was also used to confirm molecular assignment.

Preparation of the Pro:EG (1:50) mixture

The solvent was prepared by heating the mixture of L-proline:ethylene glycol (1:50 mol/mol) at 70 °C for an hour until a clear solution was obtained.

General procedure for the synthesis of thiazolo[5,4-*d*]thiazole

The Pro:EG (1:50) solvent (4.4–4.8 g) was introduced into a 50-mL round bottom flask. Then, aldehyde (2 mmol), dithiooxamide (1 mmol), and sodium metabisulfite (1.1 mmol) were sequentially added to the DES, and the mixture was stirred at 130 °C for an hour. Then, the reaction mixture was cooled to room temperature, and water (40 mL) was added to precipitate the product. The solid was filtered and washed with cold water (2 × 15 mL) followed by ethanol (5 mL).

**4,4′-(thiazolo[5,4-*d*]thiazole-2,5-diyl)bis(2-methoxyphenol**) (**TzTz 1**). Dark brown solid, 75% yield (classical heating) and 92% yield (microwave heating). m.p.: 232 °C. **^1^H NMR** (400 MHz, DMSO-*d*_6_, ppm): δH 9.83 (s, 2H, -O*H*), 7.53 (d, *J* = 2.1 Hz, 2H, *H*_Ar_), 7.44 (dd, *J* = 8.2 Hz, *J* = 2.1 Hz, 2H, *H*_Ar_), 6.91 (d, *J* = 8.2 Hz, 2H, *H*_Ar_), and 3.88 (s, 6H, -OC*H*_3_); **^13^C{^1^H} NMR** (100 MHz, DMSO-*d*_6_ ppm): δC 168.8, 150.2, 149.5, 148.6, 125.2, 120.3, 116.5, 109.8, and 56.2.

**5,5′-(thiazolo[5,4-*d*]thiazole-2,5-diyl)bis(2-methoxyphenol)** (**TzTz 2**). Dark brown solid, 70% yield (classical heating) and 75% yield (microwave heating). m.p.: 176 °C. **^1^H NMR** (400 MHz, DMSO-*d*_6_ ppm): δH 9.55 (s, 2H, -O*H*), 7.44 (m, 4H, *H*_Ar_), 7.07 (d, *J* = 9.1 Hz, 2H, *H*_Ar_), and 3.85 (s, 6H, -OC*H*_3_); **^13^C{^1^H} NMR** (100 MHz, DMSO-*d*_6_ ppm): δC 168.6, 150.8, 149.7, 147.5, 126.6, 118.4, 113.1, 113.0, and 56.2. **HRMS (ESI^−^)** *m*/*z* [M − H]^−^ calcd for C_18_H_14_N_2_O_4_S_2_: 385.032223, found 385.032235.

**4,4′-(thiazolo[5,4-*d*]thiazole-2,5-diyl)bis(benzene-1,2-diol)** (**TzTz 3**). Brown solid, 99% yield. m.p.: 344 °C. **^1^H NMR** (400 MHz, DMSO-*d*_6_ ppm) δH 7.40 (d, *J* = 2.2 Hz, 2H, *H*_Ar_), 7.31 (dd, *J* = 8.2 Hz, *J* = 2.2 Hz, 2H, *H*_Ar_), and 6.86 (d, *J* = 8.2 Hz, 2H, *H*_Ar_); **^13^C{^1^H} NMR** (100 MHz, DMSO-*d*_6_ ppm): δC 168.3, 148.9, 148.7, 145.9, 124.8, 118.2, 116.2, and 113.0.

**4,4′-(thiazolo[5,4-*d*]thiazole-2,5-diyl)diphenol)** (**TzTz 4**). Brown solid, 72% yield. m.p.: 295 °C. **^1^H NMR** (400 MHz, DMSO-*d*_6_, ppm): δH 10.20 (s, 2H, -O*H*), 7.84 (d, *J* = 8.8 Hz, 4H, *H*_Ar_), and 6.91 (d, *J* = 8.8 Hz, 4H, *H*_Ar_); **^13^C{^1^H} NMR** (100 MHz, DMSO-*d*_6_, ppm): δC 168. 7, 160.7, 149.5, 128.4, 124.9, and 116.6.

**3,3′-(thiazolo[5,4-d]thiazole-2,5-diyl)diphenol** (**TzTz 5**). Brown solid, 85% yield. m.p.: 325 °C. **^1^H NMR** (400 MHz, DMSO-*d*_6_, ppm): δH 9.93 (s, 2H, -O*H*), 7.42–7.46 (m, 4H, *H*_Ar_), 7.36 (t, *J* = 4.3 Hz, 2H, *H*_Ar_), and 6.94 (ddd, *J* = 0.9 Hz, *J* = 2.4 Hz, *J* = 8.1 Hz, 2H, *H*_Ar_); **^13^C{^1^H} NMR** (100 MHz, DMSO-*d*_6_, ppm): δC 168.7, 158.1, 150.1, 134.3, 130.7, 118.4, 117.1, and 112.5.

**2,5-bis(4-fluorophenyl)thiazolo[5,4-*d*]thiazole** (**TzTz 6**). Brown solid, 58% yield. m.p.: 181 °C. **^1^H NMR** (400 MHz, CDCl_3_, ppm): δH 8.00 (dd, *J* = 8.9 Hz, *J* = 5.2 Hz 4H, *H*_Ar_) and 7.18 (t, *J* = 8.8 Hz, 4H, *H*_Ar_); **^13^C{^1^H} NMR** (100 MHz, CDCl_3_, ppm): δC 166.8 (d, ^1^*J* = 243 Hz), 163.1, 151.0, 130.4 (d, ^4^*J* = 3 Hz), 128.6 (d, ^3^*J* = 8 Hz), and 116.5 (d, ^2^*J* = 22 Hz).

**2,5-di(pyridin-2-yl)thiazolo[5,4-d]thiazole** (**TzTz 7**). Brown solid, 66% yield. m.p.: 307 °C. **^1^H NMR** (400 MHz, CDCl_3_, ppm): 8.65 (d, *J* = 4.0 Hz, 2H, *H*_Ar_), 8.23 (d, *J* = 7.9 Hz, 2H, *H*_Ar_), 7.85 (t, *J* = 6.2 Hz, 2H, *H*_Ar_), and 7.36–7.39 (m, 2H, *H*_Ar_); **^13^C{^1^H} NMR** (100 MHz, CDCl_3_, ppm): δC 171.0, 153.4, 151.5, 149.8, 137.3, 125.2, and 120.1.

**2,5-bis(4-bromophenyl)thiazolo[5,4-d]thiazole** (**TzTz 8**). Black solid, 36% yield. m.p.: 305 °C. ^1^H NMR (400 MHz CDCl_3_, ppm): δH 7.87 (dt, *J* = 8.5 Hz, *J* = 1.9 Hz, 4H, *H*_Ar_) and 7.62 (dt, *J* = 8.6 Hz, J = 1.9 Hz, 4H, *H*_Ar_); ^13^C{1H} NMR (100 MHz CDCl_3_, ppm): δC 168.2, 151.2. 132.5, 127.9, and 125.2.

## Figures and Tables

**Table 1:** Synthesis of TzTz **1** in DES.

Entry	DES	Conversion ^1^
1	CC:Gly (1:2)	Slow (24 h)
2	CC:urea (1:2)	Slow (24 h)
3	Pro:Gly (1:2)	Fast (1 h)

^1^ Conversion followed by TLC.

**Table 2:** Optimization of temperature reaction for the synthesis of TzTz **1** in DES.

Entry	Temperature	Consumption of Vanillin ^1^	TzTz 1 Formation
1	110 °C	1 h	Yes
2	45 °C	24 h	No
3	75 °C	5 h	Yes

^1^ Conversion followed by TLC.

**Table 3:**
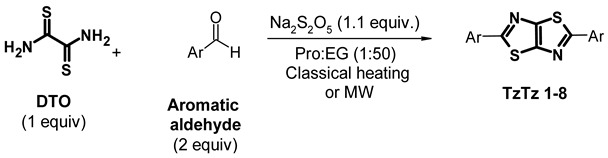
Synthesis of TzTz under optimized conditions.

Entry	Compound	Classical Heating	Yield (%)	Microwave Heating	Yield (%)	Literature
1	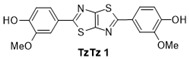	130 °C—1 h	75	130 °C—25 min	92	Pyridine, reflux, 24 h 79% [15]
2	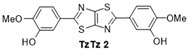	130 °C—1 h	70	130 °C—25 min	75	
3	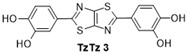	130 °C—1 h	99	130 °C—25 min	61	Nitrobenzene, 130 °C, 24 h 79% [13]
4	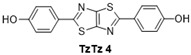	130 °C—1 h	72	130 °C—25 min	36	Pyridine, reflux, 5 h 74% [43,44] EtOH, NaOH, 80 °C, 2 h [22] Phenol, reflux, 2 h—38% [19]
5	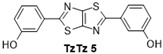	130 °C—1 h	85	130 °C—25 min	30	Excess aldehyde, phenol reflux 15 min—38% [9]
6	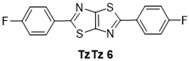	140 °C—1 h	58	130 °C—25 min	/	DMF, 150 °C, 6 h—50% [45]
7	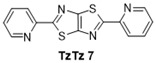	140 °C—1 h	66	130 °C—25 min	/	EtOH, Et_3_N, reflux, 48 h—58% [38] (i) Nitrobenzene, 150 °C, mw 30 min (ii) chloranil, THF, reflux, 10 min—76% [16] Nitrobenzene, 130 °C, 24 h—60% [13] Excess aldehyde, reflux, 45 min—19% [11]
8	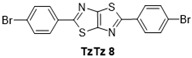	130 °C—1 h	36	130 °C—25 min	/	DMF, reflux, 3 h—27% [46]

## Data Availability

Data are contained within the article and Supplementary Materials.

## Supplementary Materials

The following supporting information can be downloaded at: https://www.mdpi.com/article/10.3390/molecules30040938/s1, Figure S1: Number of publications with thiazolo[5,4-*d*]thiazole as key word according to Web of Science. Search performed in November 2024; Figure S2: ^1^H NMR spectrum (400 MHz, DMSO-*d*_6_) of crude TzTz **1** synthesized in DMF and in DES Pro:Gly (1:2); Figure S3: ^13^C{^1^H} NMR spectrum (400 MHz, DMSO-*d*_6_) of DTO after 1 h heating at 110 °C in DMF (a) without L-proline and (b) in presence of L-proline; Figure S4: Superimposed ^1^H NMR spectra (400 MHz, DMSO-*d*_6_) of crude TzTz **1** synthesized in (a) Pro:Gly (1:4), (b) Pro:Gly (1:40), (c) Pro:EG (1:4), (d) Pro:EG (1:40) and (e) Pro:EG (1:50); Figure S5: ^1^H NMR spectrum (400 MHz, DMSO-*d*_6_) of crude TzTz **1** synthesized in EG with 5 mol% L-proline; Figure S6: ^1^H NMR spectrum (400 MHz, DMSO-*d*_6_) of L-proline alone at 0.0173M and L-proline:EG (1:50) (with the same concentration in L-proline) Figure S7: ^1^H NMR spectrum (400 MHz, DMSO-*d*_6_) of crude TzTz **1** synthesized in Pro:EG (1:50) in the presence of Na_2_S_2_O_5_; Figures S8–S23: Copies of ^1^H and ^13^C NMR of TzTz **1**–**8**; Figure S24: Experimental (red) and theoretical (black) isotopic distributions for [C_18_H_13_N_2_O_4_S_2_^−^] ion assigned after ESI (-) FT-ICR MS analysis of TzTz **2**.
