# Supplementary material for: Synthesis of Thiazolo[5,4-d]thiazoles in an Eco-Friendly L-Proline–Ethylene Glycol Mixture
Source: Molecules. 2025 Feb 18;30(4):938. doi: 10.3390/molecules30040938 (PMC11858519; doi:10.3390/molecules30040938)
Supplement: Supplementary file 1 [file molecules-30-00938-s001.zip › molecules-3462160-supplementary.pdf]

SUPPORTING INFORMATION

Synthesis of Thiazolo[5,4-*d*]thiazoles in an Eco-Friendly L-Proline-Ethylene Glycol Mixture

Thiên Thuy Trang Nguyễn, Jean-François Longevial and Stéphanie Hesse *

LCP-A2MC, Universite de Lorraine, 1 Boulevard Arago, F-57000 Metz, France
* Correspondence: stephanie.hesse@univ-lorraine.fr; Tel.: +33-3-72-74-91-80

1. Data supporting the manuscript

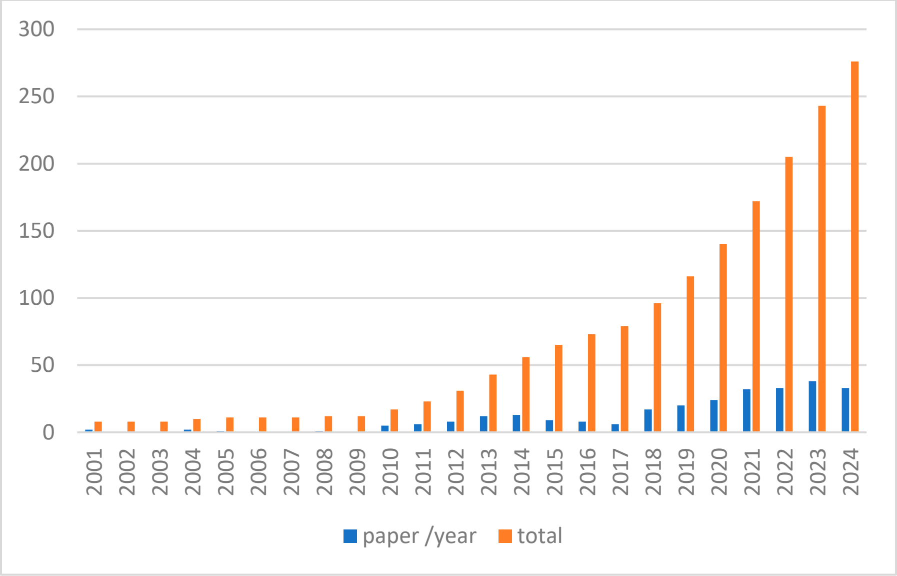

Figure S1. Number of publications with ‘thiazolo[5,4-*d*]thiazole’ as key word according to Web of Science. Search in November 2024.

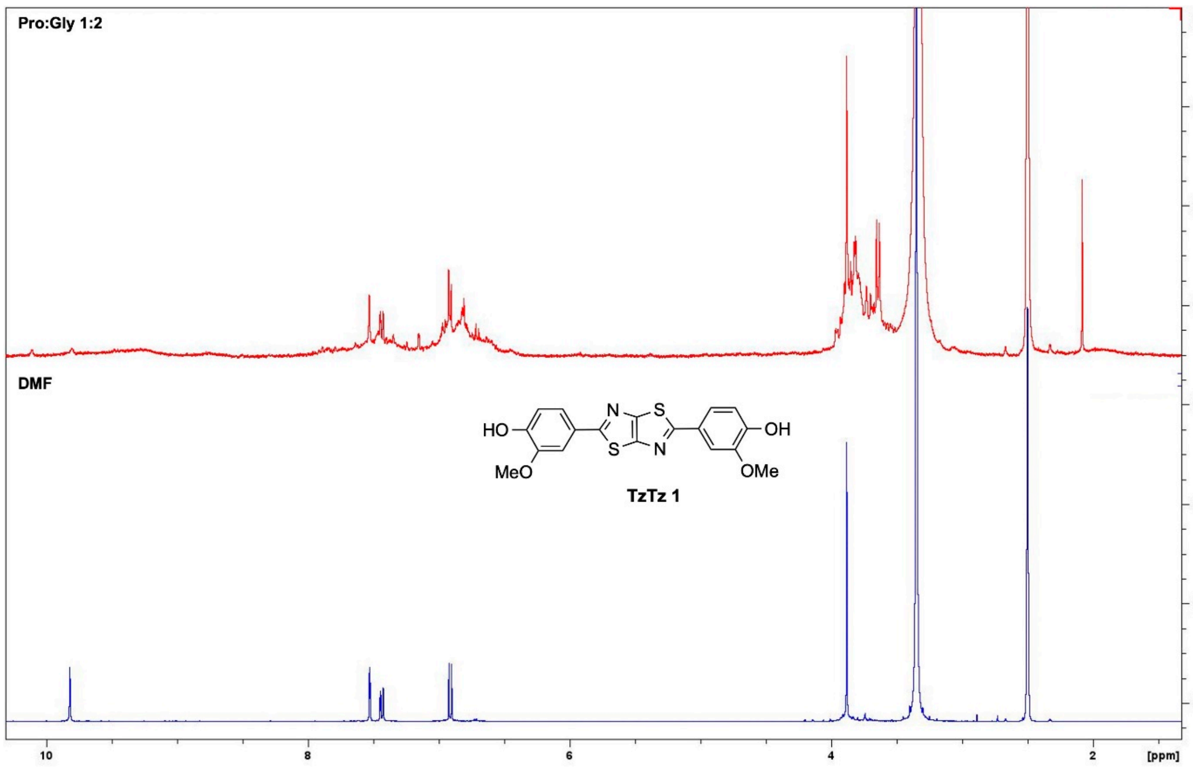

Figure S2. ^{1}H NMR spectrum (400 MHz, DMSO-d_6) of crude **TzTz 1** synthesized in DMF and in DES Pro:Gly (1:2)

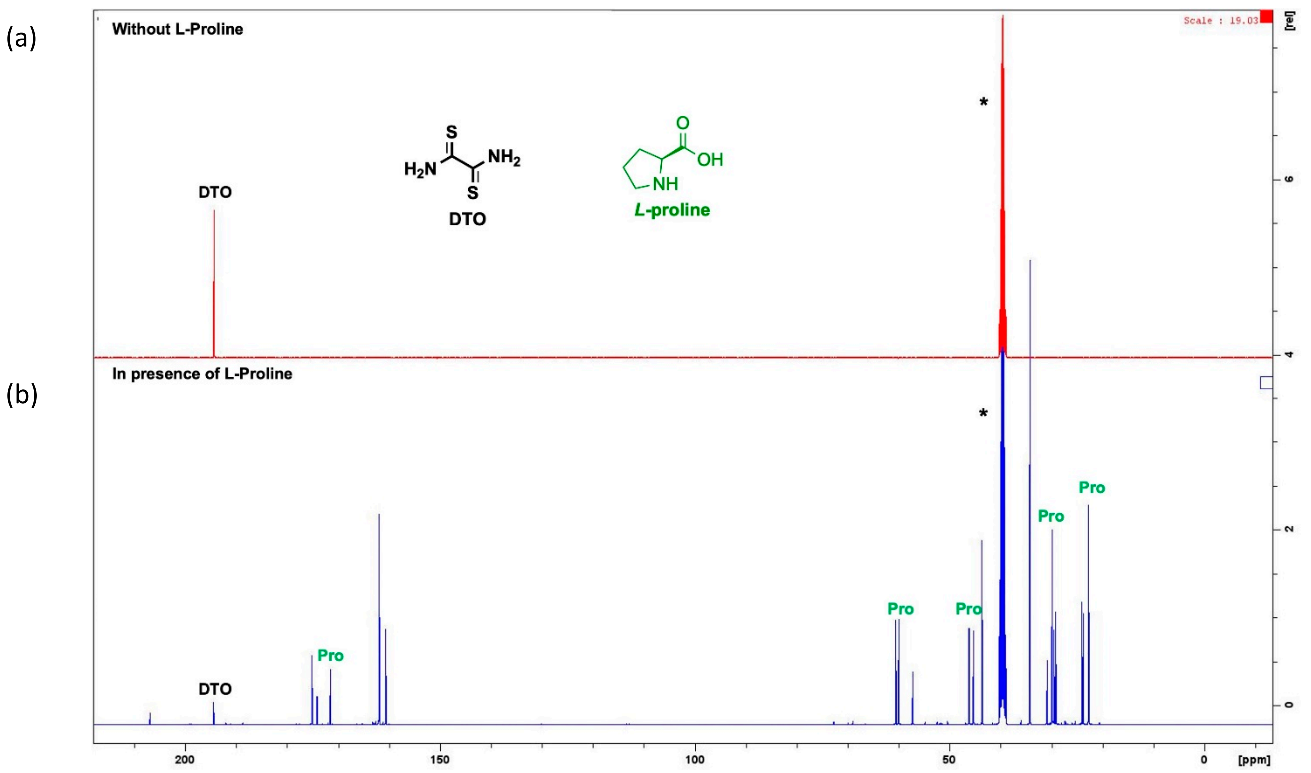

Figure S3. $^{13}C\{^1H\}$ NMR spectrum (400 MHz, DMSO-d_6) of DTO after 1h heating at 110 °C in DMF (a) without L-Proline (b) in presence of L-Proline

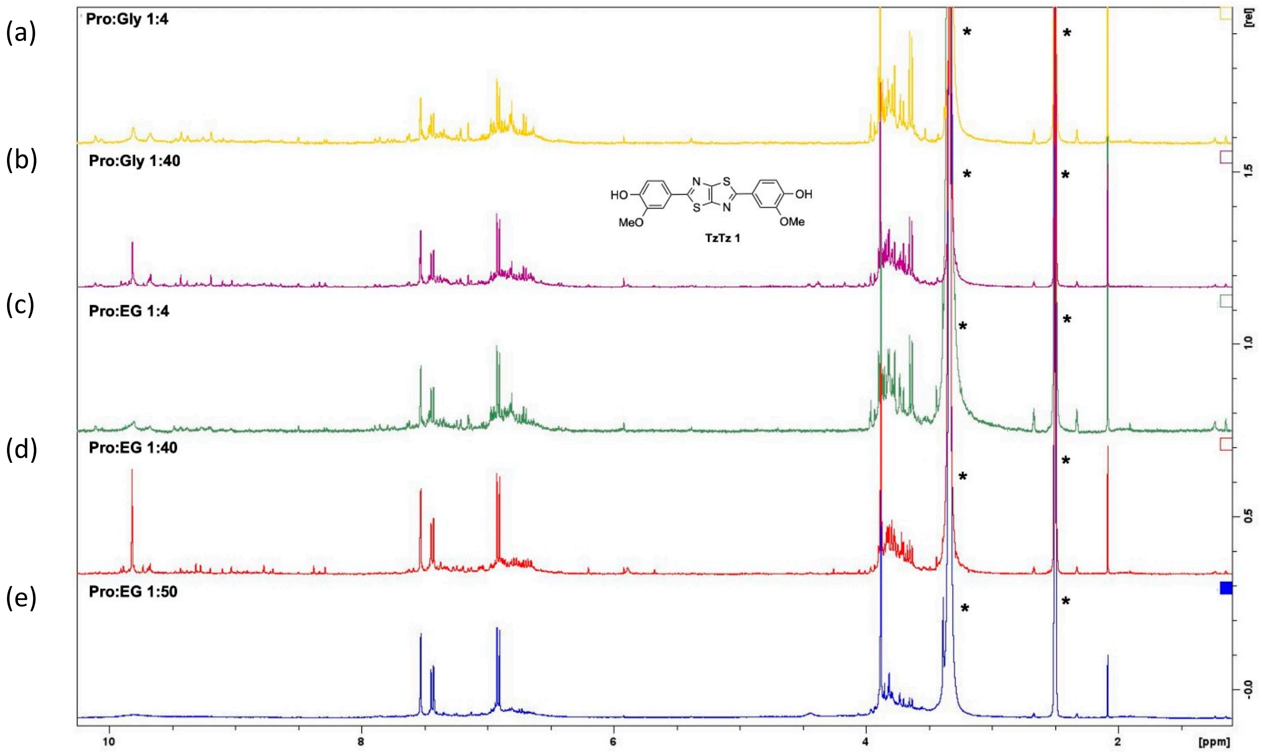

Figure S4. Superimposed ^1H NMR spectra (400 MHz, DMSO-d_6) of crude **TzTz 1** synthesized in (a) Pro:Gly (1:4), (b) Pro:Gly (1:40), (c) Pro:EG (1:4), (d) Pro:EG (1:40) and (e) Pro:EG (1:50).

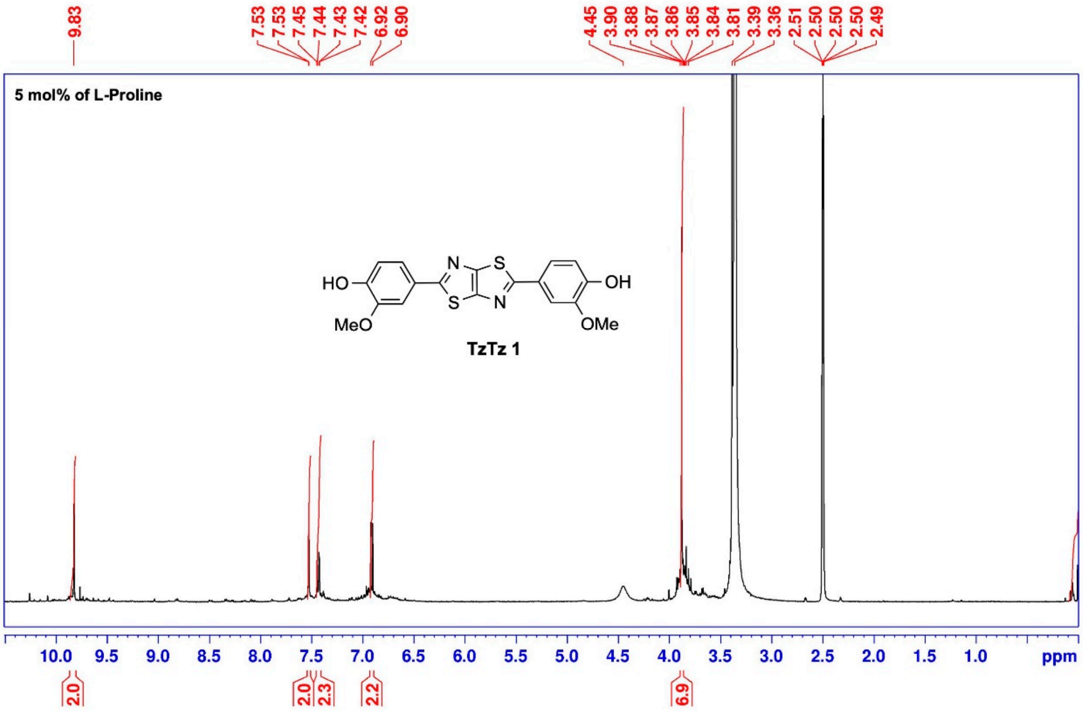

Figure S5. ^{1}H NMR spectrum (400 MHz, DMSO-d_6) of crude **TzTz 1** synthesized in EG with 5 mol% L-Proline.

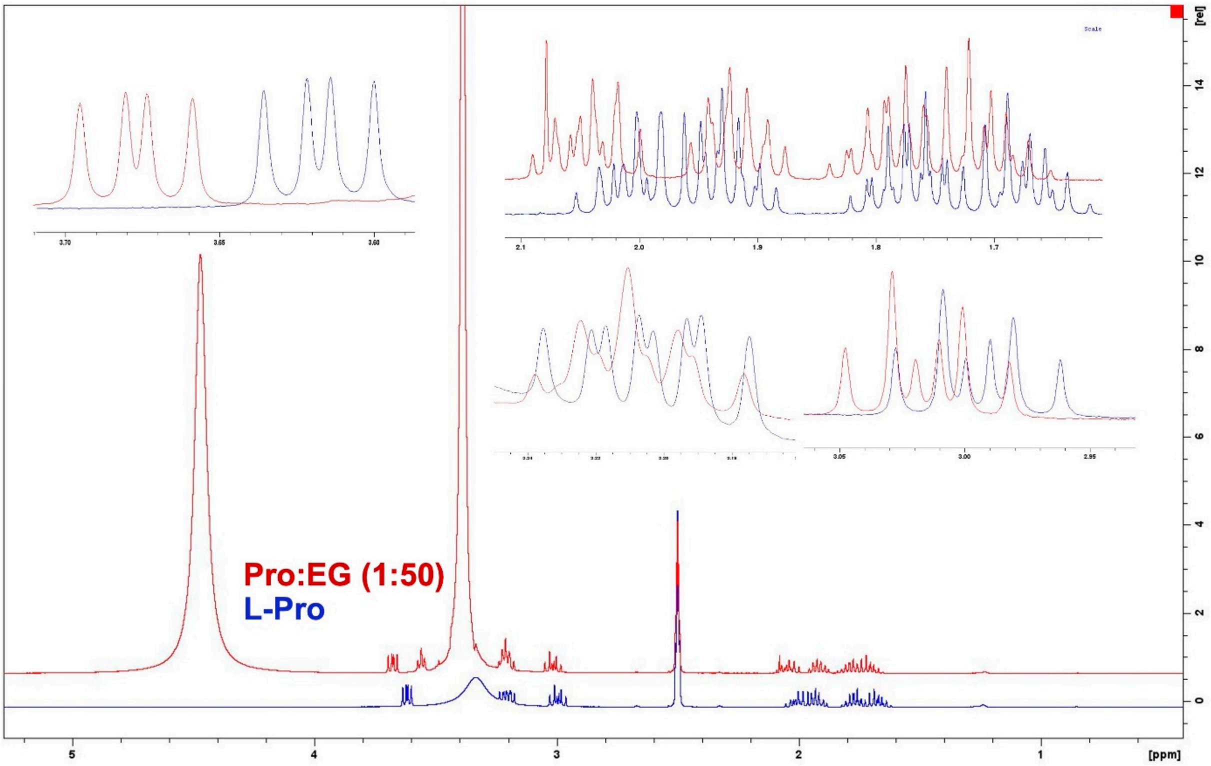

Figure S6. ^{1}H NMR spectrum (400 MHz, DMSO-d_6) of L-Proline alone at 0.0173M and L-Proline:EG (1:50) (with the same concentration in L-Proline)

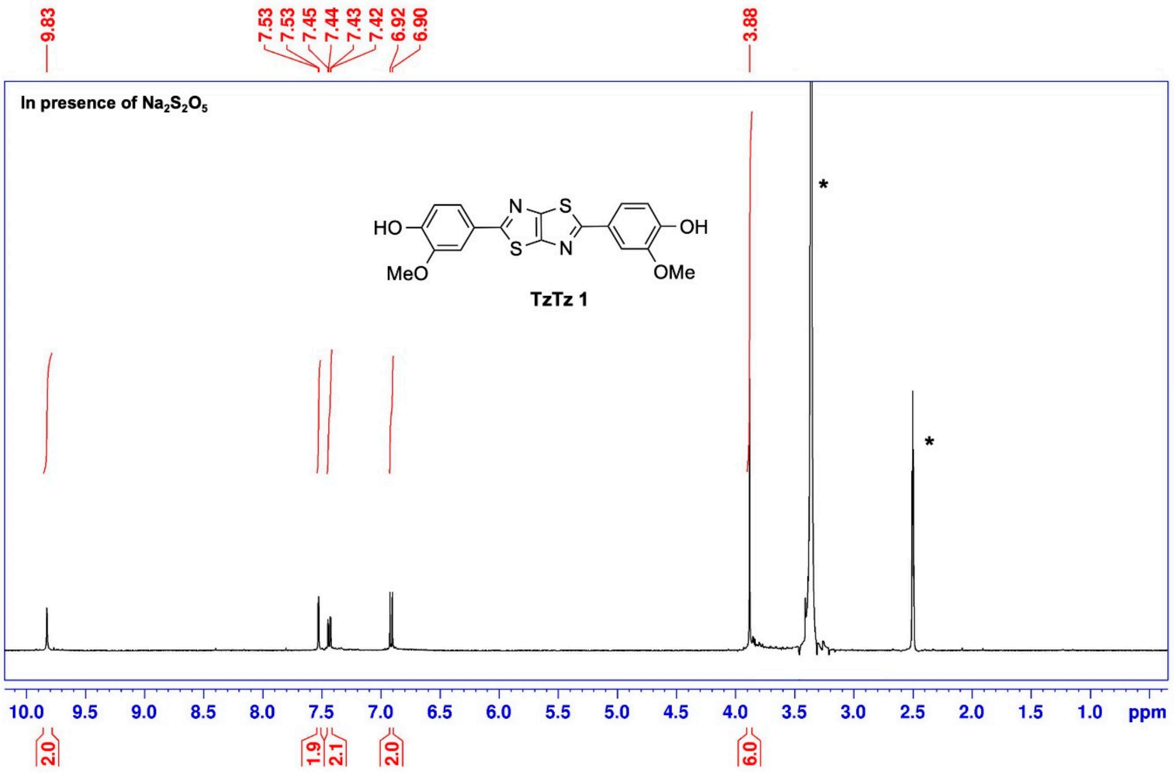

Figure S7. 1H NMR spectrum (400 MHz, DMSO-d_6) of crude **TzTz 1** synthesized in Pro:EG (1:50) in presence of $Na_2S_2O_5$

2. **^{1}H and ^{13}C NMR Spectra of TzTz 1-8**

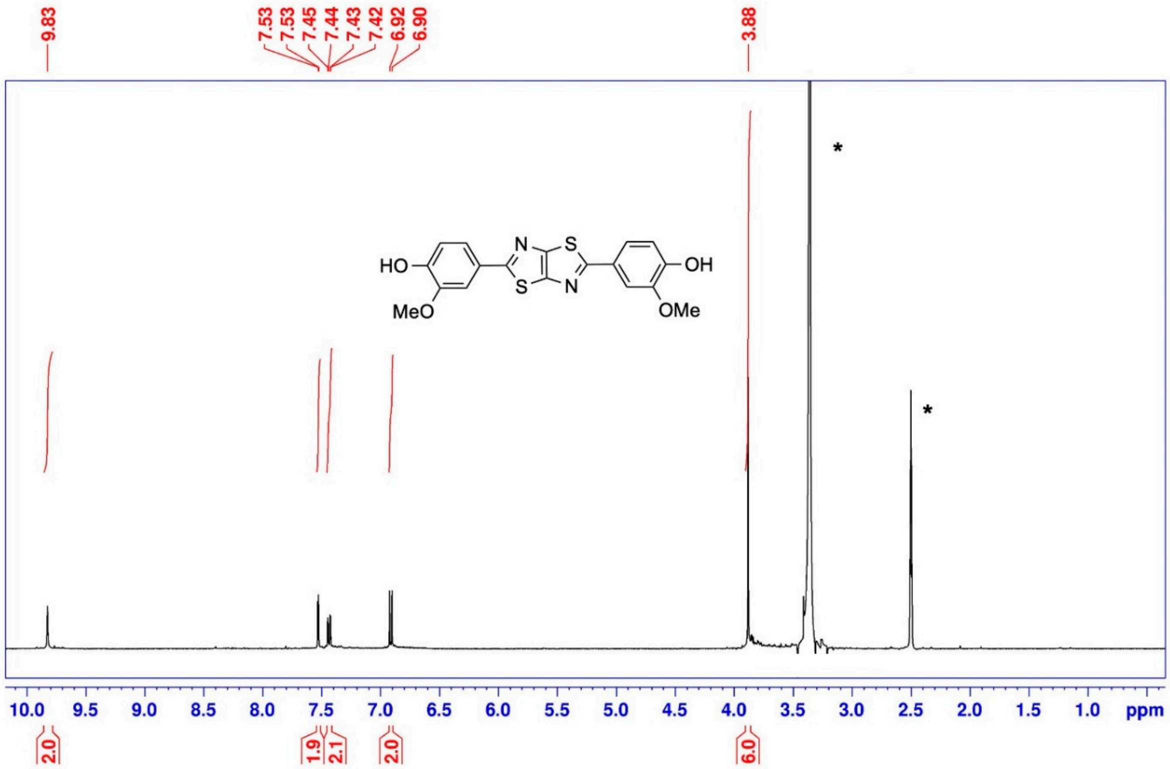

Figure S8. ^{1}H NMR spectrum of **TzTz 1** in DMSO-d_6, 400 MHz, 298 K

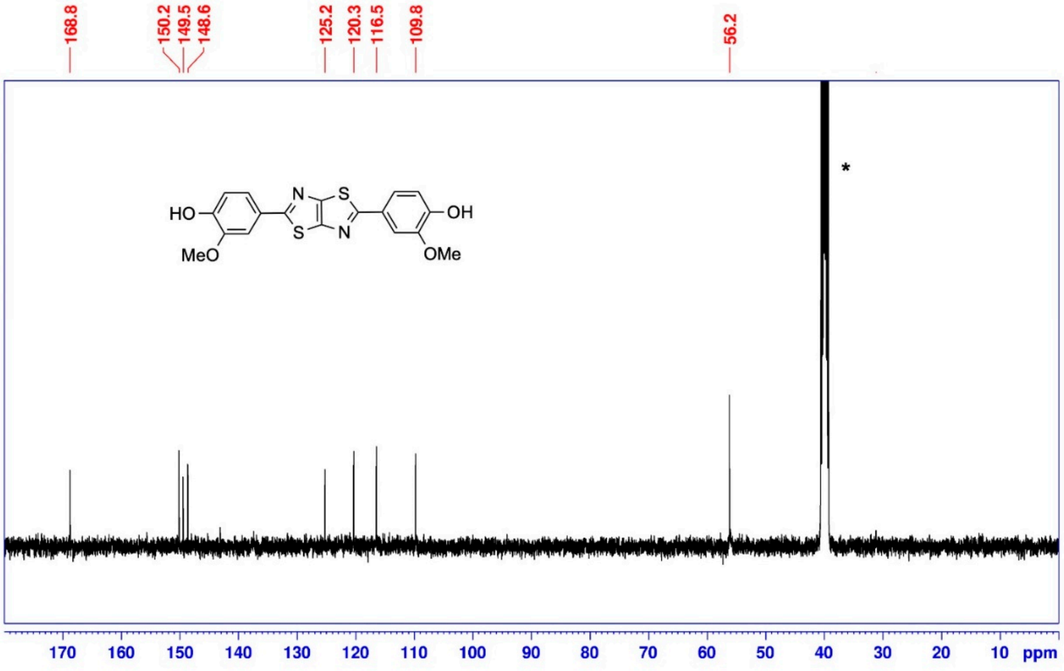

Figure S9. ^{13}C {^{1}H} NMR spectrum of **TzTz 1** in DMSO-d_6, 100 MHz, 298 K

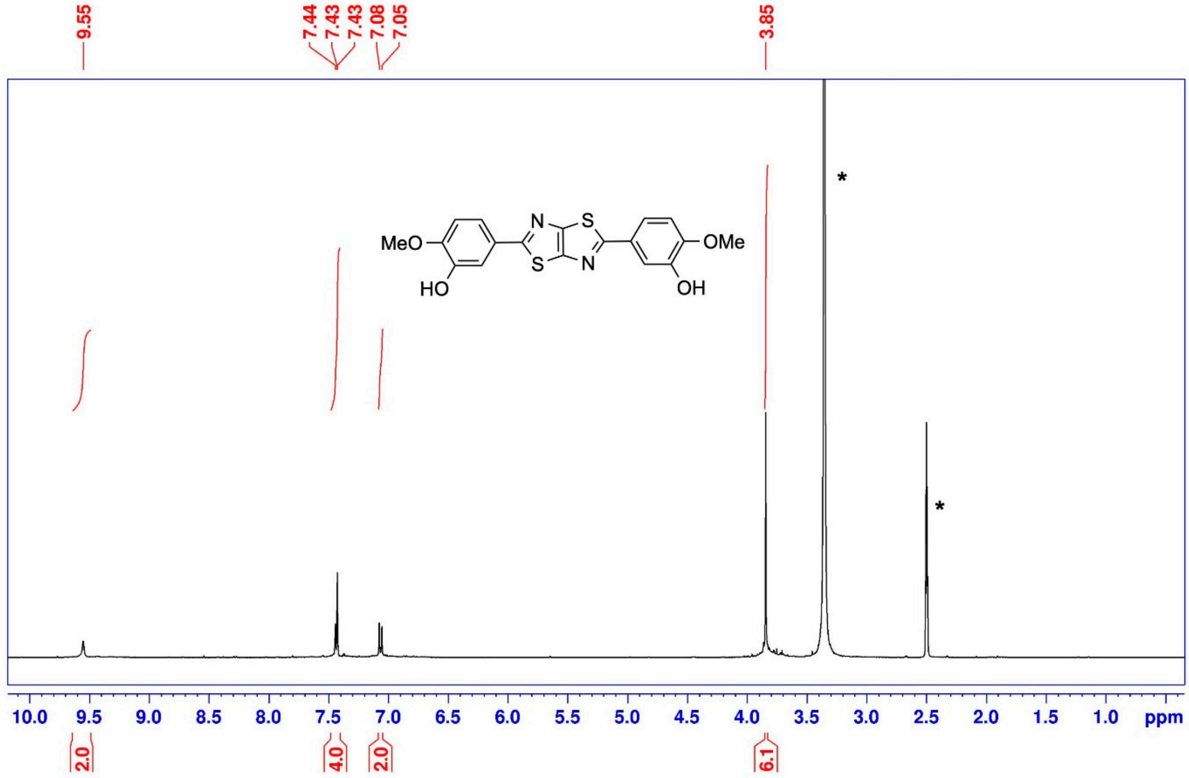

Figure S10.^{1}H NMR spectrum of **TzTz 2** in DMSO-*d*$_6$, 400 MHz, 298 K

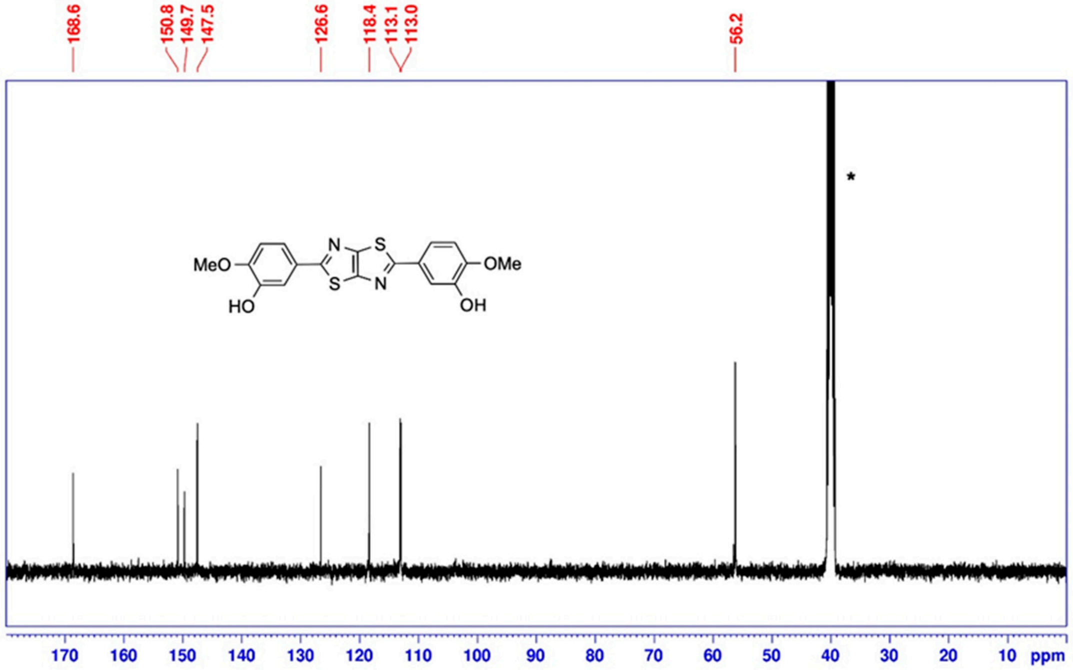

Figure S11. ^{13}C {^{1}H} NMR spectrum of **TzTz 2** in DMSO-d$_6$, 100 MHz, 298 K

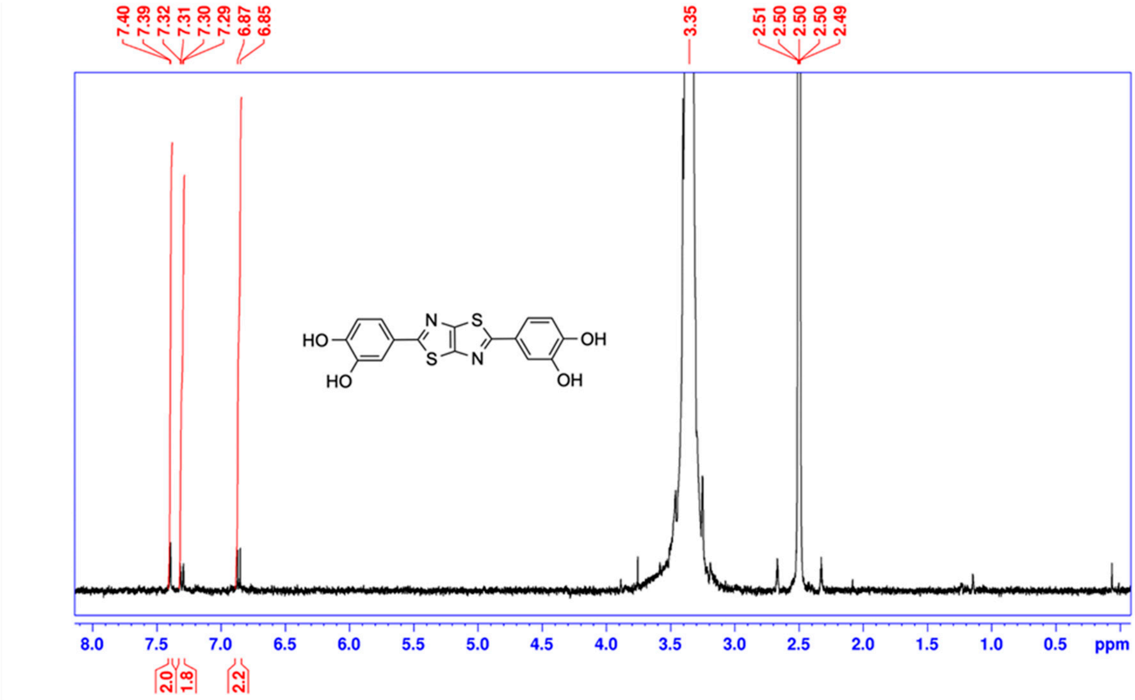

Figure S12.^{1}H NMR spectrum of **TzTz 3** in DMSO-d_6, 400 MHz, 298 K

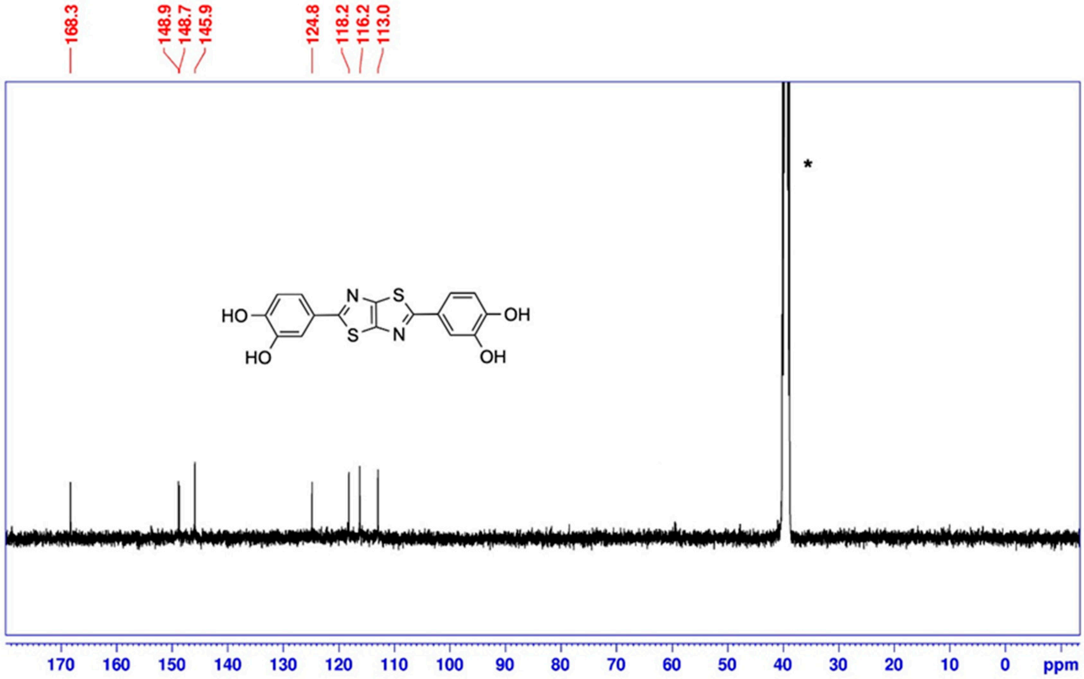

Figure S13. ^{13}C {^{1}H} NMR spectrum of **TzTz 3** in DMSO-d6, 100 MHz, 298 K

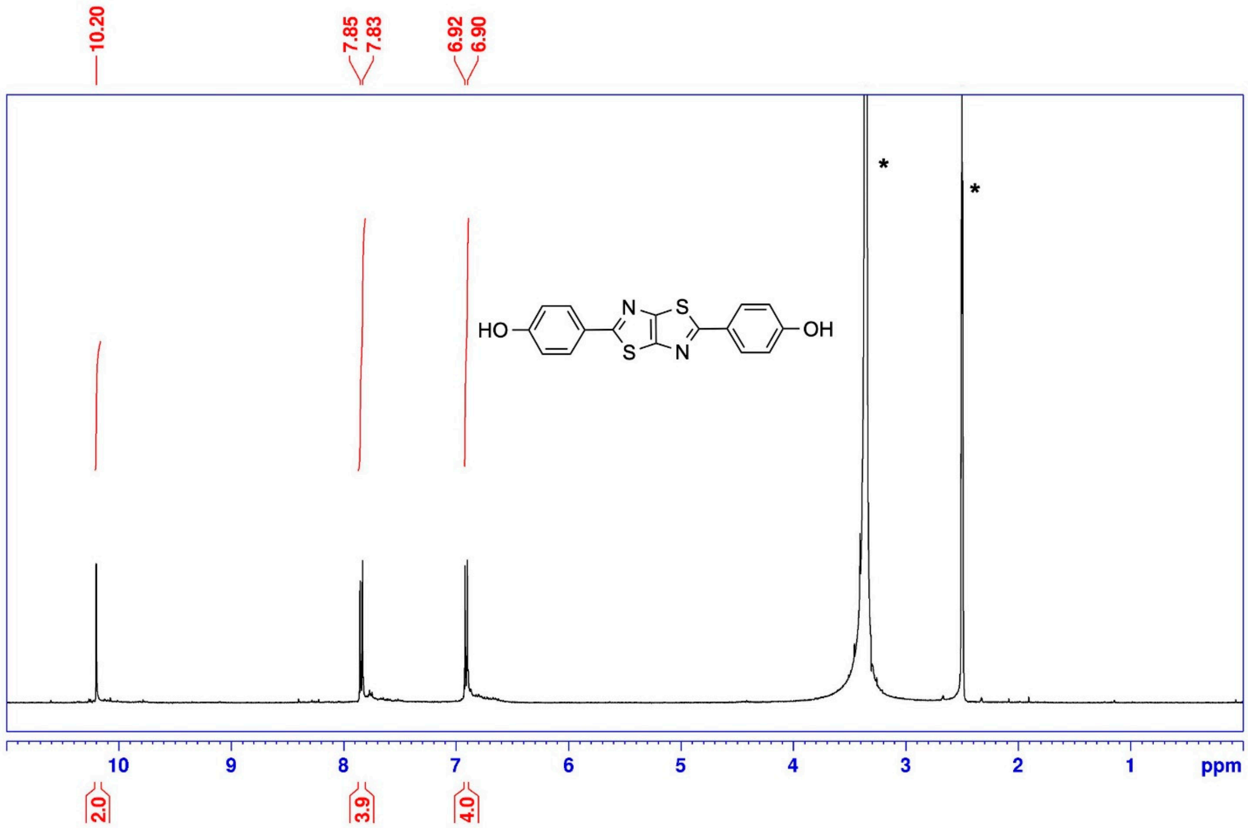

Figure S14. ^{1}H NMR spectrum of **TzTz 4** in DMSO-d_6, 400 MHz, 298 K

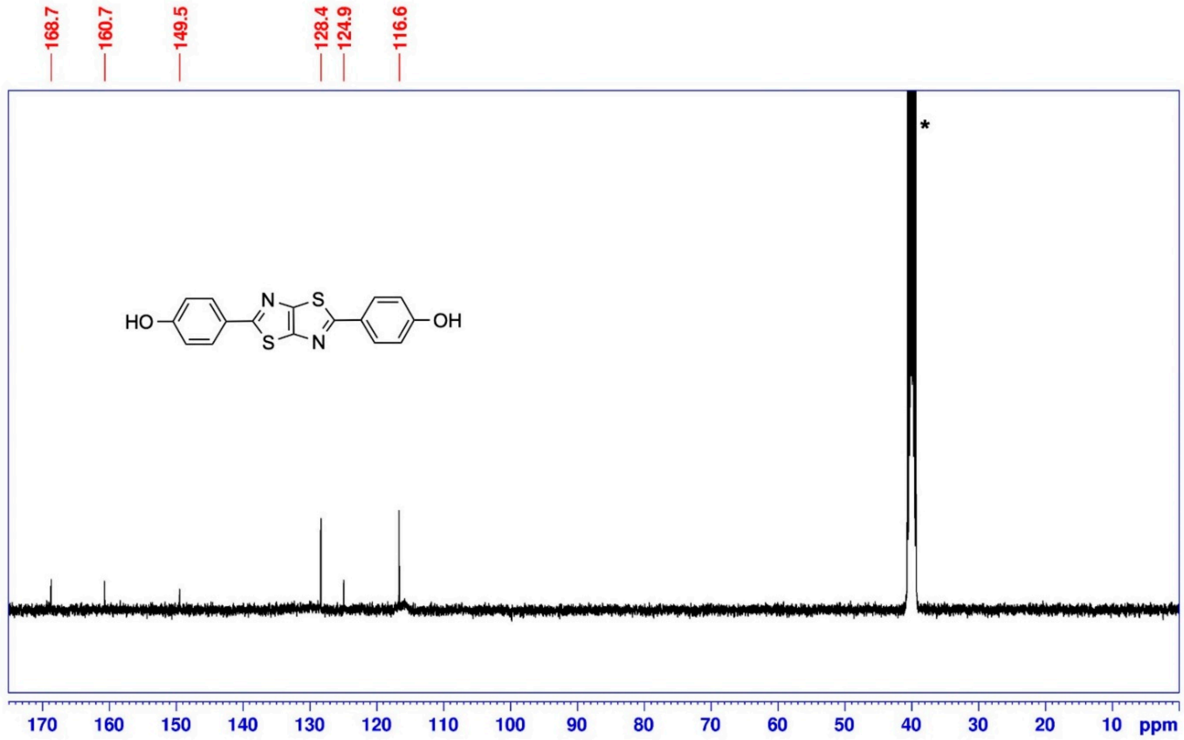

Figure S15. ^{13}C {^{1}H} NMR spectrum of **TzTz 4** in DMSO-d_6, 100 MHz, 298 K

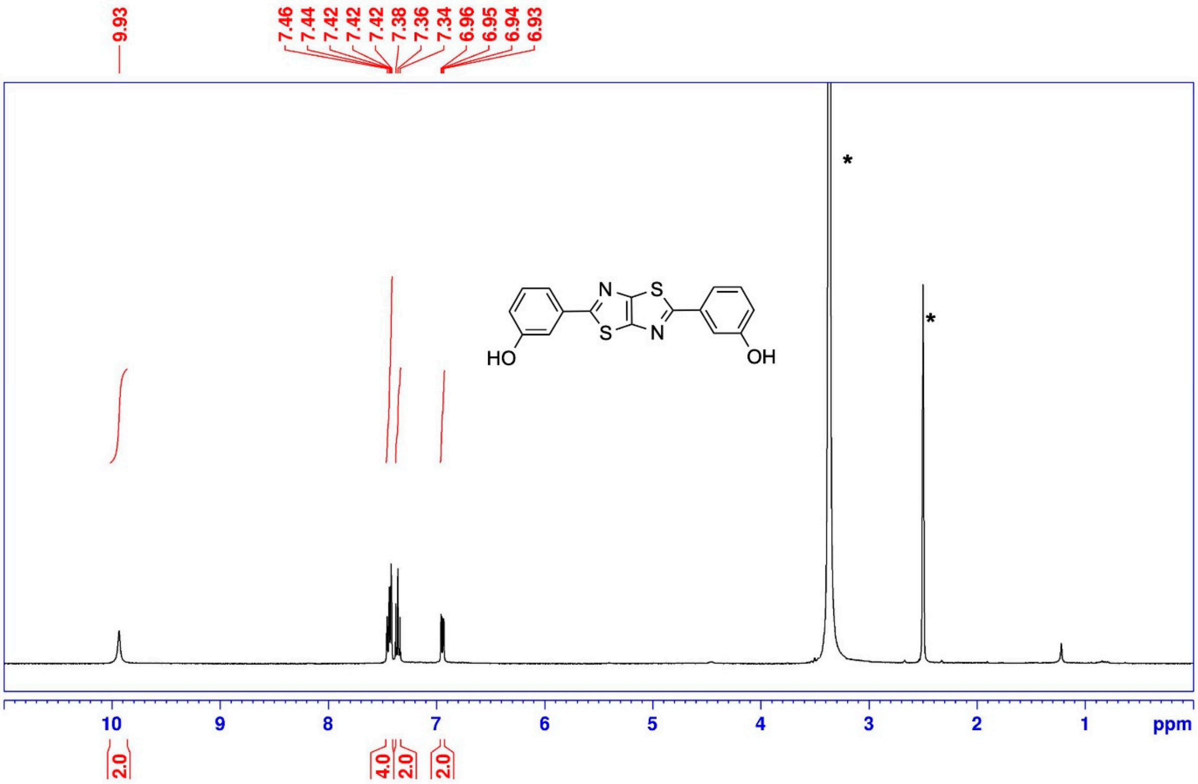

Figure S16. ^{1}H NMR spectrum of **TzTz 5** in DMSO-d_6, 400 MHz, 298 K

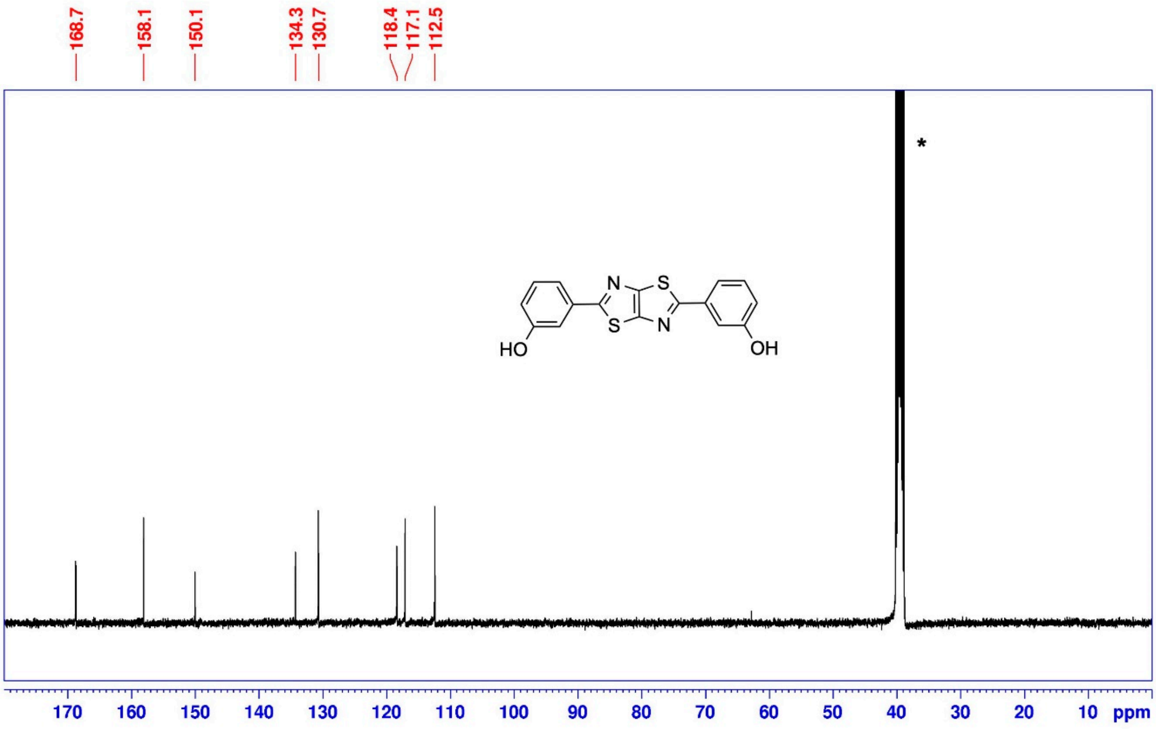

Figure S17. ^{13}C {^{1}H} NMR spectrum of **TzTz 5** in DMSO-d_6, 100 MHz, 298 K

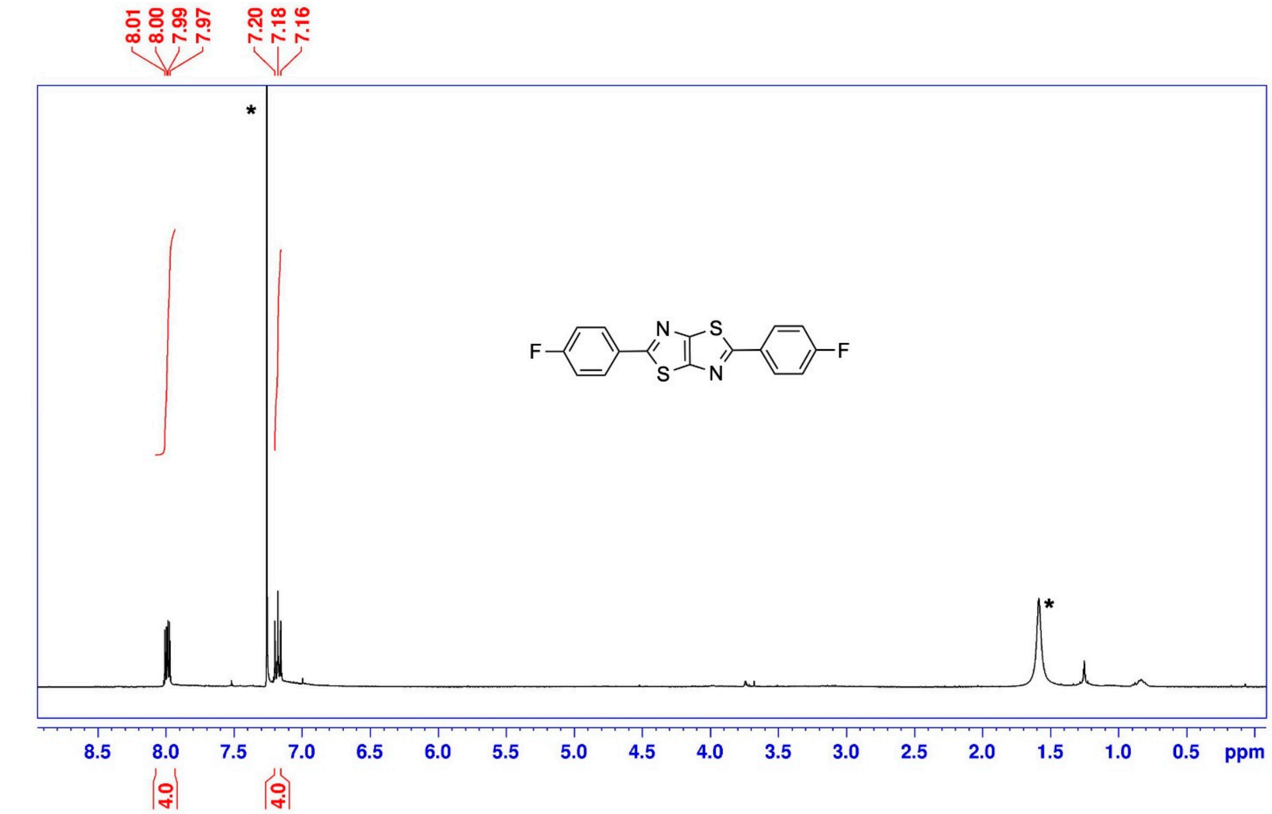

Figure S18. ^{1}H NMR spectrum of **TzTz 6** in $CDCl_3$, 400 MHz, 298 K

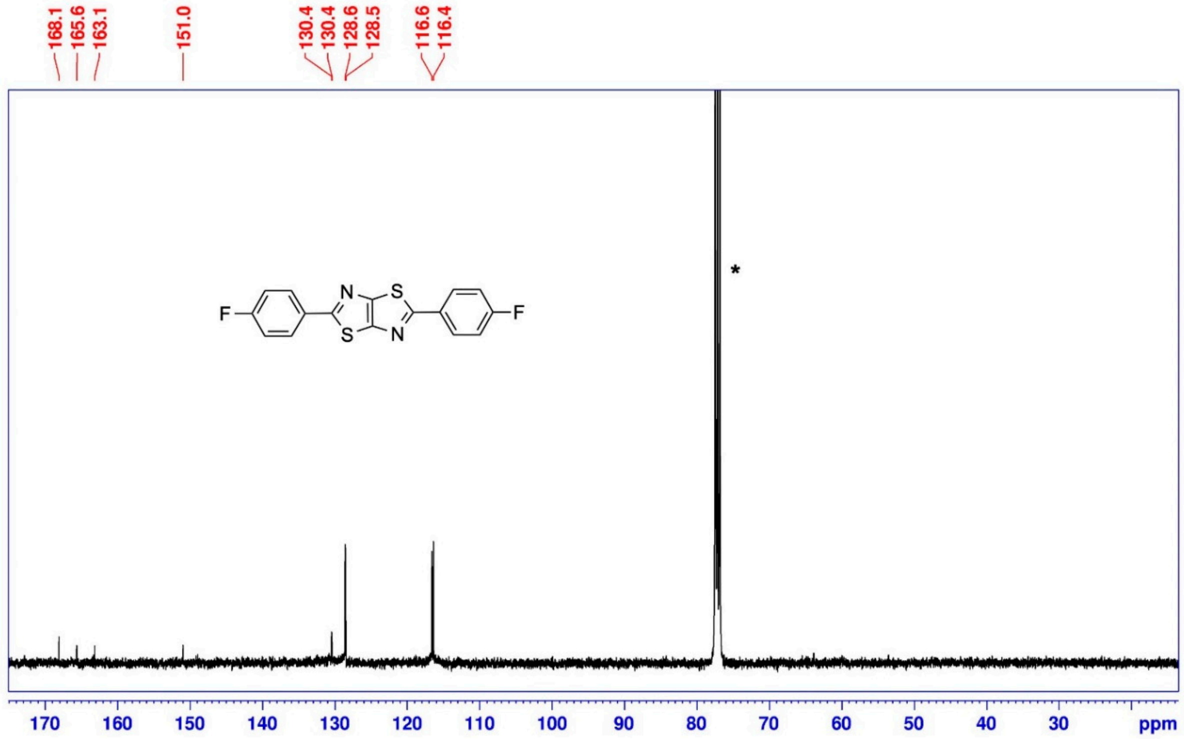

Figure S19. $^{13}C\{^{1}H\}$ NMR spectrum of **TzTz 6** in $CDCl_3$, 100 MHz, 298 K

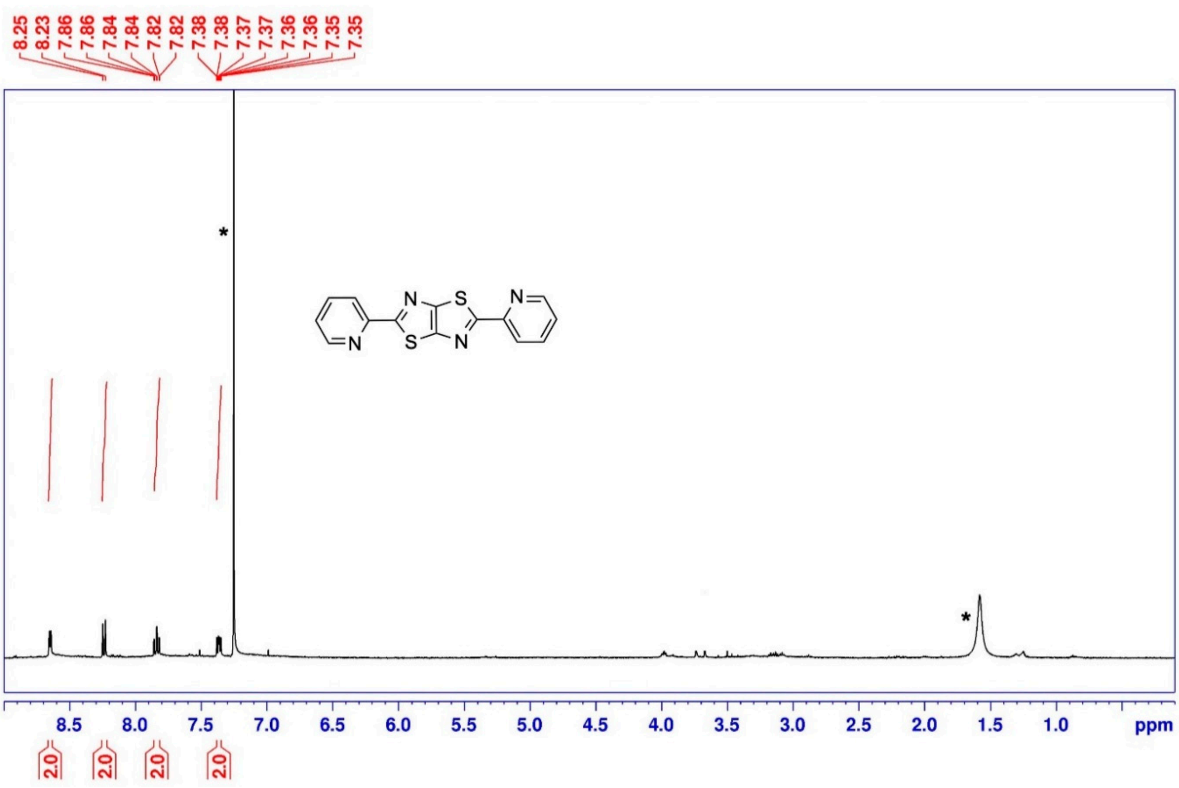

Figure S20. ^{1}H NMR spectrum of **TzTz 7** in $CDCl_3$, 400 MHz, 298 K

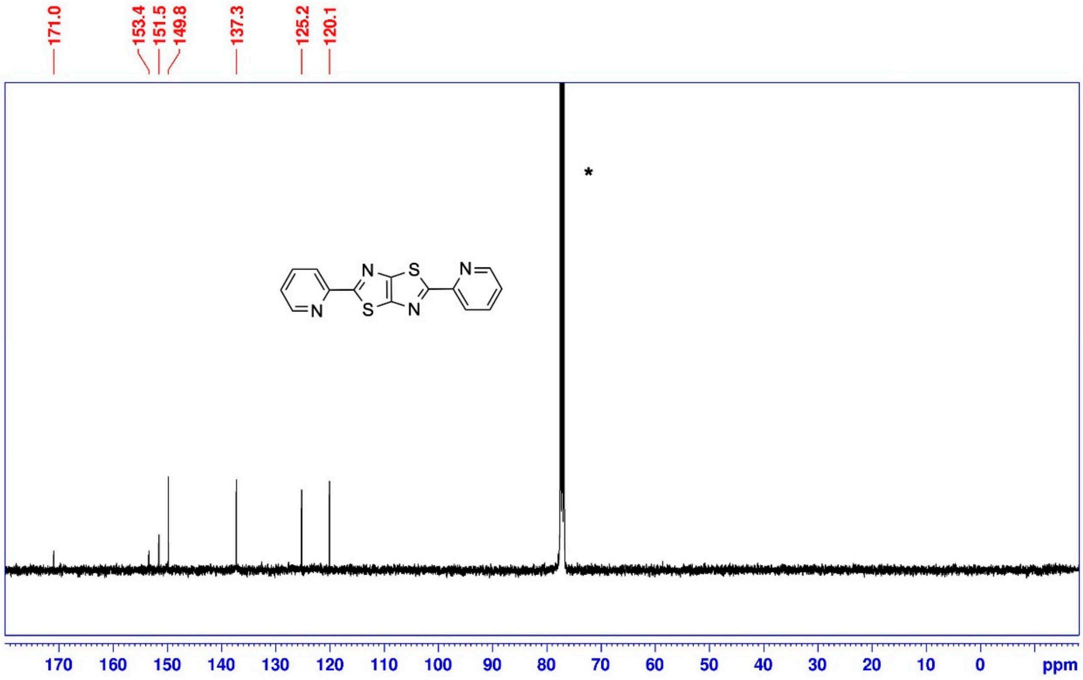

Figure S21. ^{13}C {^{1}H} NMR spectrum of **TzTz 7** in $CDCl_3$, 100 MHz, 298 K

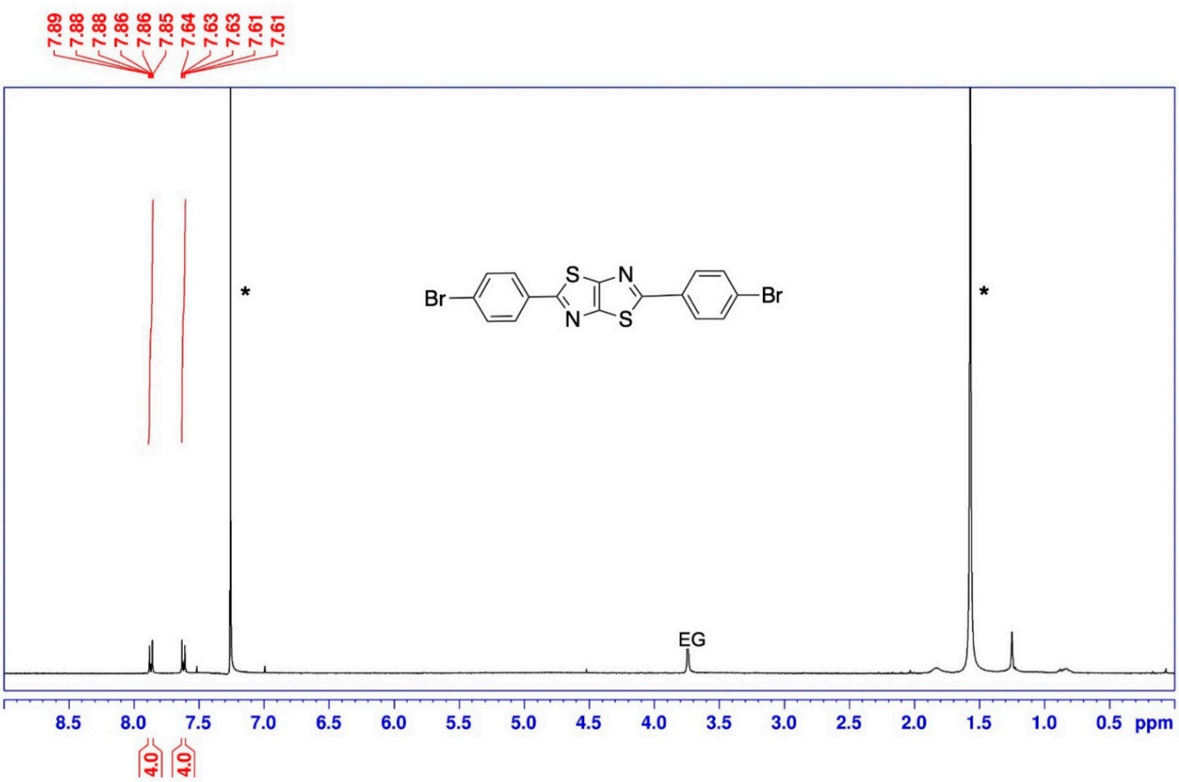

Figure S22. ^{1}H NMR spectrum of **TzTz 8** in $CDCl_3$, 400 MHz, 298 K

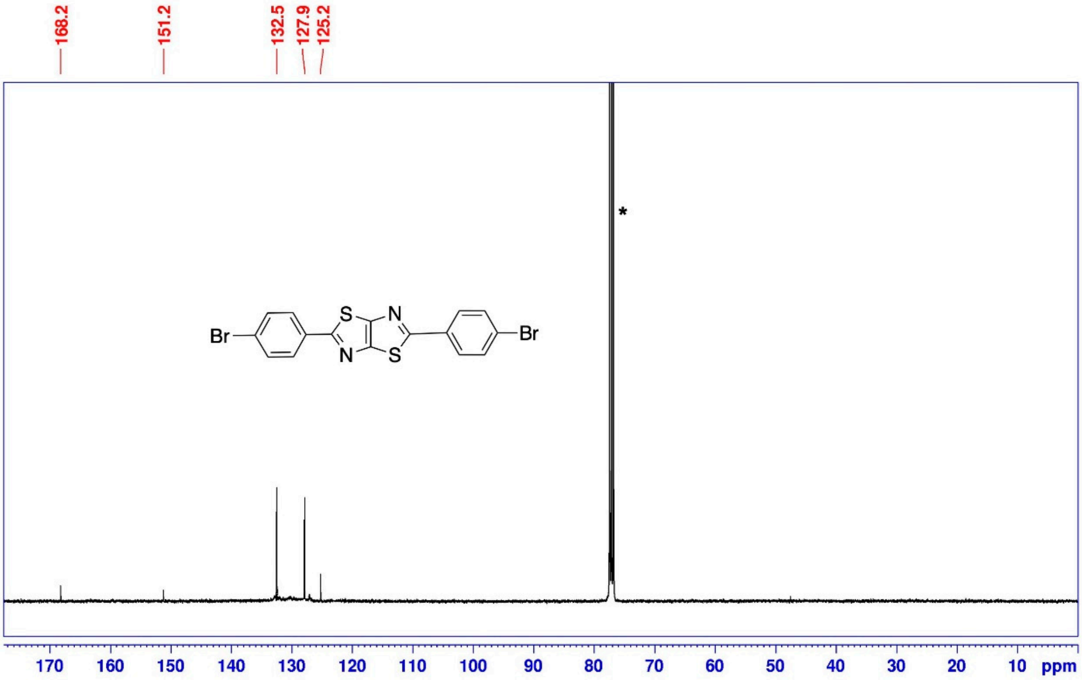

Figure S23. ^{13}C {^{1}H} NMR spectrum of **TzTz 8** in $CDCl_3$, 100 MHz, 298 K

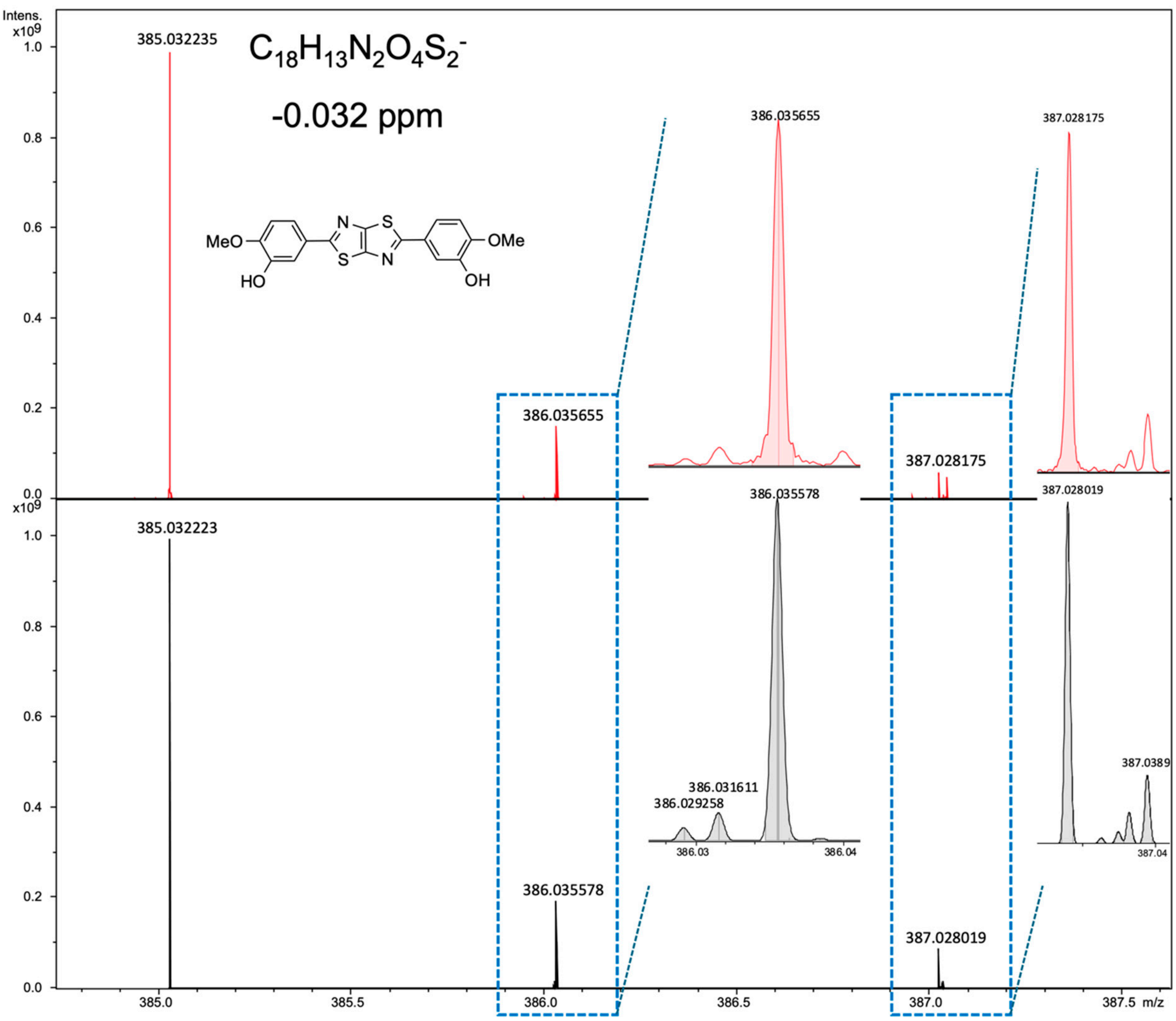

Figure S24. Experimental (red) and theoretical (black) isotopic distributions for [$C_{18}H_{13}N_2O_4S_2^-$] ion assigned after ESI (-) FT-ICR MS analysis of TzTz **2**
